# Supplementary material for: From sequence to enzyme mechanism using multi-label machine learning
Source: BMC Bioinformatics. 2014 May 19;15:150. doi: 10.1186/1471-2105-15-150 (PMC4229970; doi:10.1186/1471-2105-15-150)
Supplement: Additional file 2 — Java code of ml2db. Additional file ml2db_code.tar.gz contains the Java source code to run the multi-label machine learning experiments and save the results to database. The code’s Javadoc is included. [file 1471-2105-15-150-S2.zip › additional file 2/ml2db/utils/doc/uk/ac/standrews/utils/main/EntrezUtils.html]

EntrezUtils


JavaScript is disabled on your browser.


- Overview
- Package
- Class
- Use
- Tree
- Deprecated
- Index
- Help

- Prev Class
- Next Class

- Frames
- No Frames

- All Classes

- Summary:
- Nested |
- Field |
- Constr |
- Method

- Detail:
- Field |
- Constr |
- Method


uk.ac.standrews.utils.main

## Class EntrezUtils

- java.lang.Object
- - uk.ac.standrews.utils.main.EntrezUtils

- ---

    

  ```
  public class EntrezUtils
  extends java.lang.Object
  ```

  Utilities for Entrez services.

  Version:
  :   12 Dec 2007

  Author:
  :   Luna De Ferrari luna.deferrari-at-ed.ac.uk

- - ### Constructor Summary

    Constructors

    Constructor and Description

    `EntrezUtils()`
  - ### Method Summary

    Methods

    | Modifier and Type | Method and Description |
    | --- | --- |
    | `static java.lang.String` | `eSummaryToString(XmlNode xmlTree)` Test method to print an an Entrez eSummary xml report |
    | `static XmlNode` | `getESummaryXML(java.lang.String db, java.lang.String id)` Gets an XML tree of elements (SimpleElement) from eSummary Entrez service. |
    | `static java.lang.String` | `getNameForEntrezGeneId(java.lang.String id)` Get the entrez gene name for an entrez gene id |
    | `static java.lang.String` | `getPubMedUrl(java.lang.String pubMedIds)` Generates pubMed URL adding pubMed ids (as list separated by commas) |
    | `static void` | `main(java.lang.String[] args)` Mains for testing |
    | `static java.lang.String` | `xmlElementToString(XmlNode xml)` Test method to print to string a single Entrez eSummary xml element |

    - ### Methods inherited from class java.lang.Object

      `equals, getClass, hashCode, notify, notifyAll, toString, wait, wait, wait`

- - ### Constructor Detail


    - #### EntrezUtils

      ```
      public EntrezUtils()
      ```
  - ### Method Detail


    - #### eSummaryToString

      ```
      public static java.lang.String eSummaryToString(XmlNode xmlTree)
      ```

      Test method to print an an Entrez eSummary xml report

      Parameters:
      :   `xmlTree` -

      Returns:
      :   to string


    - #### getESummaryXML

      ```
      public static XmlNode getESummaryXML(java.lang.String db,
                           java.lang.String id)
                                    throws java.io.IOException
      ```

      Gets an XML tree of elements (SimpleElement) from eSummary Entrez
      service. Example: //
      http://eutils.ncbi.nlm.nih.gov/entrez/eutils/esummary
      .fcgi?db=gene&id=55111&retmode=xml

      Parameters:
      :   `db` - a database (for example for Entrez gene = 'gene')
      :   `id` - if database = gene > an entrez gene id

      Returns:
      :   an xml simple element

      Throws:
      :   `java.io.IOException`

      See Also:
      :   `XmlNode`


    - #### getNameForEntrezGeneId

      ```
      public static java.lang.String getNameForEntrezGeneId(java.lang.String id)
                                                     throws java.io.IOException
      ```

      Get the entrez gene name for an entrez gene id

      Parameters:
      :   `id` - the entrez gene id, eg. 1956 (EGFR)

      Returns:
      :   the entrez gene name, eg. 'EGFR' (for entrez gene id 1956)

      Throws:
      :   `java.io.IOException`


    - #### getPubMedUrl

      ```
      public static java.lang.String getPubMedUrl(java.lang.String pubMedIds)
      ```

      Generates pubMed URL adding pubMed ids (as list separated by commas)

      Parameters:
      :   `pubMedIds` - eg. 16381840,15608222

      Returns:
      :   pubMed fetch url, eg:
          http://www.ncbi.nlm.nih.gov/sites/entrez?cmd
          =retrieve&db=pubmed&list\_uids=16381840,15608222&dopt=citation


    - #### main

      ```
      public static void main(java.lang.String[] args)
                       throws java.io.IOException
      ```

      Mains for testing

      Parameters:
      :   `args` -

      Throws:
      :   `java.io.IOException`


    - #### xmlElementToString

      ```
      public static java.lang.String xmlElementToString(XmlNode xml)
      ```

      Test method to print to string a single Entrez eSummary xml element

      Parameters:
      :   `xml` -

      Returns:
      :   the string value of the xml element


- Overview
- Package
- Class
- Use
- Tree
- Deprecated
- Index
- Help

- Prev Class
- Next Class

- Frames
- No Frames

- All Classes

- Summary:
- Nested |
- Field |
- Constr |
- Method

- Detail:
- Field |
- Constr |
- Method
